# Materia Medica

**Published:** 1835

**Authors:** 


### Art. VII.—Materia Medica.

*Elements of Materia Medica and Theraputics, by Anthony Todd Thompson, M. D.; Professor of Mat. Med. and Therapeut. and of Medical Jurisprudence in the University of London, etc. etc. Second Ed. pp.* 1073.

(From a Correspondent.—Concluded.)

We propose to continue our analysis of this valuable work. We regret that the author has augmented the size of the book by the accumulation of much irrelevant matter,—thus we have ten entire pages devoted to the operation of climate upon the system, and a speculative consideration of the varieties observable in the race.

But as we have said, our purpose is not to criticise, but to give an analysis of the work.

### Section V.

*Narcotics, Syn. Anodynes, Hypnotics.*

"Some of the distinctions between this class of medicines and sedatives, have already been pointed out; but there are others that require to be noticed. *Narcotics*, strictly so called, operate as diffusible excitants, and this so decidedly, that by regulating the doses and the repetition of them, the sleep, which generally follows their administration, may be altogether prevented, and the exciting influence of the medicine only obtained: now, the effect of a direct sedative is an immediate depression of the powers of the system, without any apparent previous excitement. The symptoms of collapse which follow those of excitement, after the administration of a narcotic, are the consequence of the excitement; and, although this does not occur in the direct ratio of the degree of the excitement, yet, if it be considerable and quickly raised to its acme, the state of collapse which succeeds is proportionate; this is not the case with sedatives. The depression which they cause is the result of a peculiar action upon the nervous energy, that at once depresses it, and if the dose be large, destroys both *mobility* and *sensibility*. In a few words, a *sedative* immediately depresses the vital energies: a *narcotic* augments these energies; and this is followed by depression." p. 371.

The author, regarding narcotics in this point of view, defines them to be "medicines which in moderate doses, produce a temporary excitement, succeeded by a depression of the system, generally followed by sleep." All the narcotics do not exert an anodyne power at all correspondent to their soporific;—the influence exercised by some of them in inducing sleep, being disproportionately greater than that of alleviating pain.

In what manner is their influence exerted upon the organs of the body?

1. They act in a special manner upon the nerves of the stomach, and thence communicated to the nervous centres. The digestive powers of the stomach are lowered almost to a state of inaction by the secondary results of narcotism. The primary effect—the direct mode of action—of a narcotic upon the stomach it is impossible to conjecture. The introduction of opium into the rectum, and even its application to the skin, as well as its action on the stomach, suspends the process of chymification, should it be going on. On the intestines the impression of narcotics is to diminish their ordinary contractility, and produce constipation. Their action on the alimentary canal is modified by disease. When the mucous membrane is suffering from subacute inflammation, or is in a highly irritable state, narcotics produce thirst and dryness of the tongue, and vomiting often occurs. In irritable states of the stomach, opium, combined with tonics, aids their influence in promoting the appetite and favoring the digestive function. In ulcerations and open cancer of the mucous membrane, they allay pain and procure momentary comfort to the sufferer. In peritoneal inflammation they cause anxiety, general uneasiness, and vomiting.

2. Narcotics act upon the circulating and respiratory organs. The pulse, says our author, shortly after taking any narcotics, is in some instances small and feeble; in others full and soft; but always more or less irregular. The capillary system is particularly affected by their agency: passive perspiration, itching of the skin, and dilation of all the erectile tissues follow their use. The temperature of the body is also lowered, owing to the diminished action of the capillaries. We cannot agree with Dr. Thompson that the perspiration induced by any narcotic,—Dover's powder, for example,—is passive in its character,—that is, dependent on relaxation of the vessels; the remedy acts by a positive stimulation imparted to the secretory function of the skin. Did narcotics act by inducing passive perspiration, they would be most admissible in cases of eutonic, or exalted action. The respirations and inspirations are lessened in number, and performed in a slower manner whilst the system is under the influence of narcotism.

3. They act on the secerning system by arresting the secretions of the gastric, hepatic, and renal functions, and excite that of the dermoid tissue.

4. They exert their chief operation on the nervous system. The author involves himself in a very obvious inconsistency, in first admitting that narcotics act on the gastric nerves when taken into the stomach, and yet operate subsequently by absorption. When injected into the veins, "yet, even in this case, says he, their influence is propagated chiefly by nervous communication,—a fact demonstrated by the experiments of Messrs. Morgan and Addison, who discovered that they produce on the inner coats of the blood vessels a peculiar impression, which is conveyed to the brain along the nerves. This is also demonstrated by the rapid effects on every part of the system, often in a space of time too short for these effects to be referred to absorption; besides, it is made known that, when the dose has been so large as to be quickly followed by fatal effects, the whole quantity of the narcotic administered has been found in the stomach." p. 374.

5. Circumstances which modify the action of narcotics. These are quantity, habit, climate, and idiosyncrasy. The poisonous effects arising from an over dose of a norcotic are familiar to our readers.

Narcotics are direct, and indirect. The direct narcotics are,

*ORGANIC PRODUCTS.

a. Morphia,—combined with meconic acid, in opium:—extract of poppies:
——— with sulphuric acid, in sulphate of morphia:
——— with muriatic acid, in muriate of morphia:
——— with acetic acid, in acetate of morphia:
Bateley's sedative solution?
Black drop?
——— with citric acid, in citrate of morphia.

b. Atropha,—in the leaves of Atropa *Belladonna.*
c. Aconita,—in the leaves of Aconitum *Storchii.*
d. Conia,—in the leaves of Conium *Maculatum.*
e. Datura,—in the herbacsous part of Datura *Stramonium.*
f. Digitalia,—in the leaves of Digitalis *Purpurea.*
g. Hyosciamia,—in the leaves of Hyosciamus *Niger.*
h. Lupulia,—in the strobules of Humulus *Lupulus.*
j. Camphor,—obtained from Laurus *Camphora.*
k. Unknown principle,—contained in Rhododendron *Crysanthum;* Lactuca *Sativa,* et *Virosa;* Arnica *Montana;* Rhus Toxicondendron.

**INORGANIC PRODUCTS.

l. Ether,—*free* and *combined.*

INDIRECT NARCOTICS.

m. Tinctures of narcotics.
n. Veratria,—contained in Colchicum *Autumnale.*

MENTAL NARCOTICS.

Music; gentle friction.

Such is our author's table of this valuable class of medicinal agents. His division of narcotics into direct and indirect is grounded on a mere vague notion. Those exciting a direct impression on the nervous system, without necessarily entering the circulation, he designates as direct; those which enter the circulation before acting on the nervous system, are indirect.

Under the head of morphia and its compounds, are many valuable remarks;—more than thirty pages are occupied in the discussion. We shall endeavor to give an outline of the whole, with as little particularity of detail as is compatible with a just, yet succinct, exhibition of some very important truths.

Morphia, morphina, or morphine, is a saline body which exists in the opium in combination chiefly with *meconic acid*, but occasionally, also, with sulphuric acid, in the form of a bimeconate, and a sulphate. It is a chrystallized, white, inodorous, bitter, alkaline body. The author enters upon a rather protracted notice of the several modes of obtaining morphia, which we pass by, without repeating. Morphia combines with sulphuric, muriatic, acetic, and citric acids, and forms soluble salts, all of which are used as thereapeutical agents. The mineral acids in a concentrated state decompose morphia, but diluted with water, they readily enter into combination with it. The dose of these salts is half a grain; they are decomposed by the alkalies, and salts of the metals. The muriate of morphia is the best of these salts, affecting the head little, and scarcely displaying any exciting powers.

*Meconic acid* is the only base with which morphia has been found naturally united: has a slightly acid taste; is inodorous, and possesses no medicinal properties. The meconate of morphia, prepared by the hand of nature in the living system of the white poppy, the papaver *somniferum*, is the narcotic principle of opium. Opium, besides the meconate of morphia, contains four other principles, *narcotina*, *codeine*, *narceine*, and *meconine*:—the odor of opium depends on a volatile oil.

All the salts of morphia are decomposed and precipitated by astringent vegetable infusions and decoctions. This liability to decomposition in the salts of morphia by various salts of the metals, alkaline bodies, and vegetable infusions, lessens their therapeutical utility.

The primary action of opium is stimulant, and its agency is on the nervous system. The action of opium on the system is modified by age, sex, temperament, custom, combination, and climate. Children and women are more readily affected by the drug than adults of the other sex. The *sanguine* temperament is more easily acted on by opium than the melancholic. Custom has great power in modifying its agency. Dr. Russell, in his history of Aleppo, describes a Turk, an opium-eater in Smyrna, who took daily three drachms. The discontinuance of the habit is very difficult. The habitual use of this narcotic engenders a train of melancholy symptoms; after the excitement, subsequent to its introduction into the stomach is over, a wretched state of ennui, horror, and depression follows. When pain is present, immense quantities of the drug can be taken, without the same injurious effects which flow from its employment in health. "Dr. Chapman mentions an instance in which a wine glass-ful of laudanum was taken several times in twenty-four hours, for many months in succession, to alleviate pain in passing biliary calculi; and yet, after the patient was finally recovered, no bad effects whatever were perceptible on any of the functions. He also states that Dr. Monges and Dr. La Roche of Philadelphia had given it, in a case of cancer of the uterus, to the extent of three pints of laudanum, besides a considerable portion of solid opium, in twenty-four hours, without any effect except that of relieving pain. It is not easy to account for this effect of opium on the diseased habit; but the fact is indisputable, and it instructs how far we may proceed in administering it in cases of disease." p. 401.

Combination greatly modifies the operation of opium on the animal economy. Combined with antimonials, its diaphoretic powers are greatly increased; and it is rendered less likely to impede the other secretions. "With acids, half the usual dose will produce the effect of a full dose—a result depending on the production of a soluble salt." We entertain some doubts on the correctness of the last statement—whether opium be given with acids or alone, its action is about the same as regards its narcotic influence. Climate exerts a slight modifying power on the agency of opium. This results from the influence of climate on the vital properties—sensibility and irritability. A warm climate exalting, and a cold climate depressing, the susceptibility of the vital energies to be acted on by remedial articles.

There obtain some differences between the operation of the salts of morphia and opium. The acetate of morphia does not produce that violent propulsion of blood into the capillaries of the system, nor does it induce heat of skin, nor elevation of pulse, as is the case subsequent to the taking of opium. Opium is therefore to be preferred where we wish to stimulate the action of the heart, as occurs in depressed conditions of the vital powers. The citrate, the muriate, and the sulphate of morphia act in a manner similar to the acetate—the muriate affecting, however, the encephalon less than any of the others, its stimulant influence is obscure; its sedative very obvious. *Narcotina*, the second active constituent of opium, not being soluble in the animal juices, possesses little or no activity on the animal body.

Dr. Thompson enters upon a very minute enumeration of the various kinds and preparations of opium—these may be found in any of our late Dispensatories, we therefore pass them by, without further comment.

With reference to the remedial employment of opium, we must never lose sight of both its stimulant and sedative influence. In all inflammatory affections, the free detraction of blood must precede its use, where we wish to have the interference of its sedative or hypnotic power. A state of nervous irritation, often consequent upon febrile excitation and liberal depletion, is best relieved by a judicious exhibition of opium, in the form of a spirituous or vinous tincture; where opium, in its entire state, inflicts great distress on the patient, either in the solid or fluid form, the salts of morphia, especially the *muriate* or *acetate*, may be given. Our author regards opium as the sheet anchor of cure in rheumatism, and looks on blood-letting as only prefatory to the use of opium. Doubtless the lancet may be pushed too far in even acute attacks of rheumatic inflammation; but, as far as our experience goes, opium is not a safe remedy in this disease. Liberal purging, with tart. emetic and colchicum, promise far more in rheumatism than opium.

In confirmed phthisis pulmonalis, our author has found a mixture, consisting of one-fourth of a grain of muriate of morphia, one fluid drachm of distilled vinegar, and ten fluid drachms of the decoction of lichen Islandicus, very serviceable in allaying cough and promoting sleep, without increasing the disposition to sweating. Opium is employed in a great variety of morbid states of the system to allay irritation, subdue pain, produce sleep, and equalize the circulating mass.

"b. Atropia.—This is one of those alkaloids which the industry of our Continential brethren, in the field of science, has brought to light. Atropia, combined with the malic acid, and other constituents of the plant, is formed by the hand of Nature in the leaves, and more particularly in the seeds of the Atropa *Belladonna*. All the parts of the plant are narcotic." Perhaps, Belladonna has more claim to the title of anodyne than any other of the narcotics. The local application of the extract has been found beneficial in neuralgia.

Dr. Golles, in Hufeland's Journal, of 1825, recommends the root as a remedy in hooping cough. "I have ordered" says Dr. Thompson, "the extract in doses of one-eighth of a grain to a child of eight years of age, and gradually increased the dose to a quarter of a grain. Its power over the cough is extraordinary. It produces a state of the skin closely resembling scarlatina, accompanied with fever, suffused eye, dimness of sight, and frequently, although not always, headache. Whilst these symptoms continue, the cough remains absent; but it returns as soon as they disappear. By keeping the habit for a sufficent time, under the influence of the remedy, the period of the disease has always been greatly shortened." p. 422.

Belladonna was suggested by Habneman as a preventive of hooping cough. His directions are to dissolve three grains of the extract in f℥s. of cinnamon water, and to administer three drops of this solution twice a day to a child a year old; adding one drop for every year, until twelve be taken for a dose.

Dr. Lermann, of Torgau, has stated that, in a fatal epidemic, which occurred in that place in 1825, he tried the preventive powers of Belladonna without benefit. The extract of Belladonna exerts a dilating effect upon the pupil, by acting on the radiating fibres of the iris, when applied to the eyelids. An ointment, formed with ℨs. of the extract, and ℨvij. of lard, affords great relief in hemorrhoids, and in chordee when rubbed on the perineum: and the pounded leaves when sprinkled upon open, cancerous sores, abate pain.

c. Aconita is the narcotic principle of all the genus Aconitum. In every form of preparation, the aconite or wolf's bane, is a medicine of extreme virulence, and demands the utmost caution in its administration.

d. Conia is the narcotic principle of the conium, *maculatum*, or hemlock. This plant is a narcotic of considerable power, and may be usefully employed in all diseases connected with great irritability. In chronic rheumatism, especially in sciatica, it exerts a very beneficent effect. The dose of the powdered leaves is from gr. iij. to gr. x, that of the conia is from one-fourth to one-half grain; and that of the tincture, from m x to m xxx, in any vehicle, except one of an acid nature, which destroys its narcotic power. The extract is generally employed, but it is very liable to deterioration, and should never be used whenever a saline efflorescence appears on the surface, as it is then of no value. The dose of the extract is from gr. i, to vj. grains, but may be gradually raised to half a drachm; and when carried to this length, it should be continued for some weeks. Much comfort has also been procured by applying the extract as a topical dressing to irritable sores, whether cancerous, or otherwise.

e. Duturia. This is an alkaloid; the active principle of the datura *stramonium*, and is found in combination with malic acid in the seeds of the plant. One of the species of the datura has always been smoked in Ceylon to relieve asthma; the poorer Turks use it instead of opium; and the Chinese infuse it in beer to produce ebriety. The Arabians were early acquainted with its norcotic powers, and employed it both as a poison and as a medicine. The influence on the system of the datura *stramonium*, or Jamestown weed, closely resembles that exerted by belladonna. The plant is given in the form of extract and of tincture. The dose of the extract, if well prepared, should not exceed half a grain at first, but may be gradually carried to six grains. In small doses, it is very useful in uterine irritation, and in cases of painful menstruation, combined with Plummer's pill and Foxglove. The tincture is made by digesting for six days, and then filtering, ℨij. of the bruised seeds in fℨxvj. of alcohol. The dose is from m x to m xx. As a topical application, this tincture has been found very efficacious in neuralgia.

f. Digitalia. This saline substance is conjectured to be the active principle of the leaves of the Foxglove;—probably it is a compound substance. The determination of this point is of little moment, as the digitalis is potent enough without the isolation of its active principle. Digitalis acts upon the nervous system, producing first stimulant, and afterwards sedative effects.

This remedy should not be given where the inflammatory diathesis is present. In phthisis, it is fitted for the advanced stages of the disease, provided the inflammatory pulse has been previously reduced. M. Neuman, of Berlin, has found it an admirable remedy in chronic catarrh, when this depends on a state of erethism of the mucous membrane of the bronchi. He infuses ʒij. of the dried leaves, fℨvj. of boiling water, and gives one spoonful of this infusion every hour, until nausea, or a sense of constriction of the throat, or irregularity of the pulse follow. The medicine is then withdrawn, and again employed, in a similar manner, should the disease persist. Experience has confirmed the efficacy of Foxglove in hypertrophy of the left venticle of the heart, with or without dilation of its cavity: it diminishes the action of the diseased organ, and with this, the vertigo, pulsation in the head, singing in the ears, and other sympathetic affections of the encephalon, attendant upon this condition of that organ. The leaves of digitalis, when used, should possess a beautiful green color and odour of the fresh plant; they should be kept from the light and air. The dose is a grain of the leaves, that of the tincture, from ten to sixty drops.

g. Hyosciamia. This is an alkaloid, the active principle of henbane, hyosciamus *niger*. The hyosciamus acts in a way analogous to opium. It is regarded as peculiarly adapted to puerperal insanity. It is useful in every case of pain, particularly in neuralgia, affecting the facial nerves. Smoking the leaves in a pipe allays the pain of toothache, and the difficulty of breathing in asthma. Applied to the surface, it diminishes pain, dilates the pupil, and soothes greatly the irritation of scrofulous ulcers and cancerous sores. The tincture is the best form of exhibiting henbane—dose, from fifteen to sixty drops; that of the extract is gr. ii, to gr. xvj; but it has been carried gradually to gr. lx. It should not be prescribed in combination with alkalies, nor with lime water, as these destroy its narcotic power.

h. Lupulia is a peculiar compound principle, spontaneously formed in the strobules of the hop, the humulus *lupulus*. It was first brought into notice by Dr. Ives, of New York. The lupulia is a weak narcotic — its dose is from ten to thirty grains.

Under the heads of the other narcotic substances, we find nothing worth analysis until we reach indirect narcotics.

Tinctures of Narcotics. "There can be no doubt, that the alcohol employed aids the introduction of the narcotic principle into the system, and enables it to be more directly applied to the nervous centres." We are incapable, we confess, of comprehending the above assertion. The stomach surely is amply endowed with nervous power, to be acted on by narcotic tinctures, without the clumsy hypothesis of the introduction of the tincture into the blood. Where we wish to soothe pain, and to stimulate at the same time, the tincture is the most eligible form of a narcotic—but when we seek for the attainment of the anodyne effect exclusively the extract, or the article in substance, is to be employed.

n. Veratria. This alkaline principle is found in the seeds of the veratrum sabadilla, the roots of veratrum *album*, and in the colchicum *autumnale*. It is a very acrid, stimulating substance.

Colchicum is an efficacious and valuable medicine. It was employed by the ancients under the name of Hermodactyllus; and as such was sold in England, by the druggists in the time of Turner, the herbalist. Alexander Trallianus, a Greek physician, first recommended it in gout. Paulus Ægineta extols it as a *purgative* in pains of the joints; and it was, after this period, very generally employed, until some circumstances again threw it out of use, when it was only occasionally resorted to as a diuretic. The surprising effects of an empirical French remedy, the eau medicinale again brought colchicum into notice, and few medicines are now so generally employed. Colchicum, consequently veratria, exerts a double action on the living system—a local stimulant influence and a narcotic sedative effect; which is probably the result of the prior stimulant action. It operates chiefly on the duodenum; exciting powerfully the excretory ducts of the liver and pancreas, producing copious bilious stools, diminishing febrile action, and allaying pain. It is chiefly from its operation on the bowels, that its beneficial effects in gout and rheumatism result. Colchicum, nevertheless, is generally supposed to exert some specific influence in gout and rheumatism." p. 446.

The wine made of the dried bulb is the best preparation—the dose is from fifteen to sixty drops repeated, every eight hours.

"Mental Narcotics. Our passions, medically considered, are phenomena which produce decided effects on our corporeal frames—frequently causing diseases, sometimes curing them. Those persons who are endowed with great nervous sensibility, are, for the most part, powerfully effected by mental pleasures and pains: and, therefore, when the higher orders of society, and men of intellectual acquirements, are affected by mental diseases, it is sometimes necessary to employ mental Narcotics, if the material fail in procuring repose."

The author remarks thus on the agency of sounds as productive of a subduing effect upon mental excitation: "It is a well known law of the system, that a variation of a stimulant impression renews the excitement in such a manner as to be much less likely to be followed by collapse than when there is a repetition of the same impression. To illustrate this, I need only refer to the effect of listening to the gurgle of the mimic cataract of some mountain rill, or to that of any small waterfall: how decidedly is felt the influence of the sound, disposing to sleep. It is a common observation, that a dull sermon is a good soporific; but I am of opinion, that it is less the dullness of the matter of the sermon, than the monotonous manner in which it is read, which produces this effect. Now, music operates, in the first instance, on this: in the second, by the associations which it engenders. If it be slow and plaintive, the impressions are longer continued, less varied, and therefore, more soporific; and along with use must also take into account the sedative influence of all depressing passions; and the melancholy, which is the effect of plaintive music, is one of these. This power of music is much increased, also, by the period of the day, and the situation in which the listeners are placed. In some peculiar cases of insanity, therefore, where music is advisable as a soporific, the evening is the best period for trying its effects: and this period is chosen, also, for the further reason, that when sleep is induced, there is much less likelihood of its being disturbed, than if it occurred in the day. In the application of this narcotic, the chief difficulty arises in determining the character of the individuals, the nature of the attack, and the kind of music best fitted for the occasion. In some, it should be such as may withdraw the mind of the patient from old associations, and turn his ideas into an entirely new channel: in others, it will prove most salutary if it recall his mind to former habits, and spread before the imagination the scene of past hours, in which domestic happiness reigned undisturbed in the bosom of the sufferer. Under such circumstances, the cloud which has settled upon the mind may be happily dispersed, and reason restored to her seat.

The effects of gentle and slow friction in producing a narcotic result, are referrible to the principle, the repetition of an agreeable impression on the nervous system. I have witnessed the powerful influence of gentle friction, in producing sleep, in many instances. In cases of pain, in particular, gentle friction produces a considerable soothing effect, by transferring the attention from the seat of pain to the mild and agreeable impression of the friction ; and this is still more powerful, if to this impression be joined sound, which, although operating on a distinct sense, yet, by the combination, is powerfully soporific; thus, the patting of an infant on the back, whilst at the same time the nurse hums a monotonous tune, is almost sure to procure sleep. I could adduce many instances of the excellent effect of such narcotics." p. 449.

SECTION VI.

*Antispasmodics.*—These are such medicines as allay irregular muscular contraction. The chief circumstance distinguishing antispasmodics from narcotics and tonics is, that the former is not followed by insensibility to impressions which results from the use of narcotics, and that they act more quickly than tonics; they are more powerful than narcotics, more rapid than tonics, in repressing inordinate muscular motion. The author indulges in a strain of subtle speculation on the modus agendi of this class of remedies, which we will not notice any further, as such refined views conduce little to a just practical conception of the curative powers of remedies.

A table of Antispasmodics is given—the principle articles are musk, castor, oil of amber, valerian, assafœtida, galbanum, tonics, narcotics, and the mental antispasmodics, fear, and abstraction.

We find nothing of interest in our author's views of this class of medicines, except his remarks on the Mental Antispasmodics. Speaking of *fear*, he remarks, "I recollect a striking instance of its sanative influence in hooping cough, kept up by the habit. The patient, a young boy, was threatened with the application of a large blister, it was not applied, but merely placed within his view; nevertheless, the dread of it completely removed the cough. Boerhave also cured epilepsy in a whole school, by displaying, at the moment of the expected attack, a red hot poker, which he threatened to poke down the throats of those who should have a fit." The agency of abstraction as an antispasmodic, is thus brought forward. "A man has his shoulder luxated; various ineffectual attempts are made to reduce it, owing to the spasm which has supervened, and which is maintained by the attention of the patient being directed solely to the part; abstract the attention by any means, the spasm instantly yields, and the head of the humurus instantly slips into its socket. It is unnecessary to pursue the subject farther: there is no doubt that both fear and sudden abstraction of attention may be successfully employed as antispasmodics; but, in admitting this, we must, at the same time, confess that great discrimination and judgment are requisite to determine the circumstances under which their employment is applicable." p. 465.

### Section VII.

Tonics—Syn. *Corroborants.* These are substances which restore strength to the body when it is weakened. They impart vigor to the muscular system, tone to the digestive apparatus, and increased energy to the circulating powers; they also prompt the capillaries to a better performance of their appropriate action, such as secretion, nutrition, and absorption, and induce an abatement of morbid nervous susceptibility.

The principal tonics are cinchonia, quinia, piperina, gentiana, quassina, bitter extractive, arsenic, tinc. bismith, iron, mineral acids, cold bathing, exercise, friction; the mental tonics are hope, confidence, travelling, and amusement.

Cinchonia is the active principle in two officinal cincona barks, in which it is united with *kinic acid*, forming a *kinate*. These barks are the cinchona *lancifolia*, and c. *oblongifolia*. Cinchonia, when separated from the kinic acid and combined with the sulphuric, or muriatic, or acetic acids, is very soluble, and eminently displays tonic effects. Cinchonia is obtained, likewise, from the cusparia bark. This is an excellent aromatic tonic in convalescence from fevers, dysentery, and diarrhæ; and whilst it increases appetite, and restores tone, it never oppresses the stomach. The dose of cusparia is from gr. v., to ℈j.

Quinia. This salt is more bitter than cinchonia, and combined with sulphuric acid forms an affective tonic. The officinal cinchonia barks, yielding quinia, are the c. *cordifolia*, and c. *oblongifolia*. Dr. Elliotson gives the sulphate of quinine to the extent of gr. x, in intermittent fever, just before the paroxysm. The best mode of prescribing it is to dissolve two or three grains in an infusion of the confection of roses, filtered,' and acidulated with dilute sulphuric acid, and to add f℥i. of tincture of orange peel, which covers the extreme bitterness of the solution. The bitterness of the sulphate is covered by aromatic bitters, such as orange peel, and cascarilla.

Piperina does not deserve to be ranked among the pure tonics—it is rather a stimulant. The dose is from gr. viij., to gr. x, every fourth hour, in the apyrexia of ague.

We find nothing particularly interesting until we come to the author's reflections on arsenic. Speaking of the arsenite of potassa, he remarks, that it "has been given, with benefit, in those cases of chronic rheumatism which assume an intermittent type: it has also proved occasionally useful in symptomatic epilepsy, chorea, and other spasmodic affections, as well as tic douloureux and cephalalgia. I have had much experience of its efficacy in lepra and some other cutaneous diseases, when given in conjunction with large doses of the pure alkalies and with conium." p. 517.

Of the sub-nitrate of bismuth, the author says, "this preparation is a powerful tonic, particularly useful in that state of stomach which produces *pyrosis.* In this complaint, the paroxysms commence with a sense of constriction and pain at the stomach, gradually subsiding after the eructation of a large quantity of either insipid or acidulous watery fluid. The subnitrate, in doses of from griij. to grx., in combination with grj. of opium, gives almost immediate relief, and affords a more decisive evidence of the primary action of tonics on the stomach, than any medicine of this class. It has been erroneously stated to be beneficial in cholera." p. 523.

"With regard to the medicinal powers of the salts of iron, the natural chalybeates are of eminent service in all cases requiring tonics: their primary effect is displayed on the digestive organs; whence their influence is propagated, rousing the nutritive faculty in every part of the body: they augment the power of the secretory system: and, by the moderate but permanent nature of the impression which they impart to the nerves, increase the tone and general vigor of all the functions. Something is undoubtedly due to the circumstances connected with drinking mineral waters at their source:—when the cares and anxieties of life have been left behind, in the smoky alley and crowded street, when hope and confidence and amusement lend their invigorating aid to the tonic influence of the salutary fountain. But tone follows the use of iron in all its forms, and therefore its preparations are employed in every disease connected with relaxation or debility, particularly of a chronic kind: dyspepsia, hysteria, amenorrhœa, leucorrhœa, scrofula, and chronic catarrh, are a few of the catalogue which chalybeates are calculated to benefit." p. 535.

"Sulphuric acid, when properly diluted, is a tonic of very considerable power. In cases of immoderate perspiration—as, for instance, in the hectic of phthisis pulmonalis—it is the appropriate remedy; and in combination with aromatics, it removes many of the urgent symptoms of dyspepsia; and it is altogether a tonic of the highest value when judiciously administered. When given to women who are suckling, it acts powerfully on the system of the infant, causing gripings, and frequently convulsions, although it has never been detected in the milk of the mother."

Of the verity of the last averment respecting its inducing infantile disease, we entertain serious doubts. When any medicine disturbs the regular and healthful operations of the maternal system, the secretion of the lactiferous tubes will partake of the effect of such abnormal action, and the child be sickened by this perverted secretion. Mental emotions in the mother often engender colic, and other affections, in the infant.

Our author, in speaking of nitric acid, says that he has frequently administered it holding in solution the bichloride of mercury, and that he never experienced any bad results from the combination, but that beneficial consequences will arise from this conjunction, in many cases of disease, especially of the surface of the body. Muriatic acid is likewise a good tonic. In malignant ulcerated sore throat, such as is frequently epidemic in London, both internally used and in gargles, combined with tincture of capsicum, and infusion of roses, this acid has been found an efficacious remedy. Cold bathing, exercise, friction, cheerful mental emotions, act as tonics, and are to be employed by the skilful physician with great effectiveness in many cases of morbid action.

### Section VIII.

*Astringents.*—These the author divides into those which exert a tonic influence on the system:—acids, and metallic salts, as allum, sulphate of iron, sulphate of zinc, etc., and astringents which exert a sedative influence such as sugar of lead, and cold. Dr. T. is confident that *Ruspini's Styptic,*

which he views as a very valuable astringent, consists of gallic acid, a small proportion of sulphate of zinc and of opium, dissolved in a mixture of alcohol and rose water.

The chapters on errhines, expectorants, and sialagogues, contain little or no matter of interest. Emetics and cathartics are well disposed of, and deserve careful perusal. Our notice of the work has extended beyond our original design; we must, therefore, come to a conclusion of this article. Dr. Thompson has omitted entirely the consideration of anthelmintics, although invermination often is productive of disease, and should claim at our hands, as practical physicians, some consideration. J. P. H.

